# Assessing the Accuracy of an Artificial Intelligence-Based Segmentation Algorithm for the Thoracic Aorta in Computed Tomography Applications

**DOI:** 10.3390/diagnostics12081790

**Published:** 2022-07-23

**Authors:** Christoph Artzner, Malte N. Bongers, Rainer Kärgel, Sebastian Faby, Gerald Hefferman, Judith Herrmann, Svenja L. Nopper, Regine M. Perl, Sven S. Walter

**Affiliations:** 1 Department for Diagnostic and Interventional Radiology, University Hospital Tuebingen, Eberhard Karls University Tuebingen, 72076 Tubingen, Germany; christoph.artzner@med.uni-tuebingen.de (C.A.); malte.bongers@med.uni-tuebingen.de (M.N.B.); judith.herrmann@med.uni-tuebingen.de (J.H.); regine.perl@med.uni-tuebingen.de (R.M.P.); 2 Siemens Healthineers, 91301 Forchheim, Germany; rainer.kaergel.ext@siemens-healthineers.com (R.K.); sebastian.faby@siemens-healthineers.com (S.F.); 3 Department of Radiology, Brigham and Women’s Hospital, Harvard Medical School, 75 Francis Street, Boston, MA 02115, USA; ghefferm@gmail.com; 4 Viral Immunobiology, Institute of Experimental Immunology, University of Zürich, Winterthurerstrasse 190, 8057 Zurich, Switzerland; nopper@immunology.uzh.ch; 5 Department of Radiology, Division of Musculoskeletal Radiology, NYU Grossman School of Medicine, 660 1st Ave, New York, NY 10016, USA

**Keywords:** spiral computed tomography, dimensional measurement accuracy, artificial intelligence, software, thoracic aorta

## Abstract

The aim was to evaluate the accuracy of a prototypical artificial intelligence-based algorithm for automated segmentation and diameter measurement of the thoracic aorta (TA) using CT. One hundred twenty-two patients who underwent dual-source CT were retrospectively included. Ninety-three of these patients had been administered intravenous iodinated contrast. Images were evaluated using the prototypical algorithm, which segments the TA and determines the corresponding diameters at predefined anatomical locations based on the American Heart Association guidelines. The reference standard was established by two radiologists individually in a blinded, randomized fashion. Equivalency was tested and inter-reader agreement was assessed using intra-class correlation (ICC). In total, 99.2% of the parameters measured by the prototype were assessable. In nine patients, the prototype failed to determine one diameter along the vessel. Measurements along the TA did not differ between the algorithm and readers (*p* > 0.05), establishing equivalence. Inter-reader agreement between the algorithm and readers (ICC ≥ 0.961; 95% CI: 0.940–0.974), and between the readers was excellent (ICC ≥ 0.879; 95% CI: 0.818–0.92). The evaluated prototypical AI-based algorithm accurately measured TA diameters at each region of interest independent of the use of either contrast utilization or pathology. This indicates that the prototypical algorithm has substantial potential as a valuable tool in the rapid clinical evaluation of aortic pathology.

## 1. Introduction

An aortic aneurysm is defined as a progressive dilatation of the aorta due to the weakening of the vessel wall. It is associated with aortic dissection and rupture, and thus represents a potentially life-threatening pathological process [1,2,3]. The aorta consists of two main segments, the thoracic and abdominal aorta. The thoracic aorta is further anatomically subdivided into the root, ascending segment, aortic arch, and descending segment [2]. A thoracic aortic aneurysm (TAA) affects at least one of these aortic segments [2]. In the general population, the prevalence of TAA is approximately 0.3% with an incidence between 6–16.3 per 100,000 people/year with a peak incidence in males >65 years [1,4,5]. However, due to its lack of associated symptoms, the true epidemiology of TAA is difficult to accurately quantify; consequently, true incidence and prevalence are likely underestimated.

Patients with an aortic aneurysm are typically free of clinical symptoms. As a result, TAA is often incidentally visualized on computed tomographic (CT) images obtained for different clinical indications [1,2,4,6]. The feared sequelae of TAA are symptomatic, however, and include acute “tearing” back pain in the case of acute dissection with various secondary effects depending on the extent of aortic branch involvement, as well as severe acute-onset chest pain in the setting of rupture. These acute aortic events result in mortality rates as high as 21–22%, often resulting in death before medical treatment can be rendered [4,6,7].

The non-pathological diameter of the thoracic aorta varies as a function of age, sex, and body size [2]. Despite this variation, surgical repair of the thoracic aorta is recommended when the following indications are present: (1) aortic diameter exceeds 5.5 cm, which carries an increased risk of dissection or rupture of 3% annually and increases to 7% annually if the diameter exceeds 6 cm [2,4,6,8]; (2) if the aneurysmal growth rate is greater than 0.5 cm/year; and (3) if the patient becomes symptomatic [2,7]. Surgery may be warranted at smaller diameters for patients with genetic syndromes which carry an increased risk of aortic pathology, including Marfan and Ehlers–Danlos syndromes.

Manual measurement of the aortic diameters can be time-consuming and may be inaccurate due to inter-reader variability, depending on the specific plane and location where the reader is measuring [1]. Consequently, there exists a clinical need in which AI-based tools can be leveraged to increase the speed, accuracy, and inter-rater reliability of aortic diameter measurement [1,9,10].

The purpose of this study was to evaluate the accuracy of a prototypical artificial intelligence (AI)-based algorithm for automated segmentation and diameter measurement of the thoracic aorta using computed tomography. Specifically, we hypothesize that the prototype will achieve both accurate anatomic segmentation along the course of the thoracic aorta and accurate determination of the aortic diameter at each location. Thus, the application strength of the prototypical algorithm could be as a screening tool in exams not focused on the aorta.

## 2. Material and Methods

This was a single-center, HIPAA-compliant, IRB-approved retrospective study conducted with a waiver of informed consent.

### 2.1. Study Population

Images from 123 consecutive patients imaged over the course of four days (14–18 February 2020) were included in this study. All patients received a clinically indicated CT scan of the thorax using a 3rd generation dual-source CT. Patients were excluded if: (1) the thoracic region was not scanned (*n* = 53), and (2) if patients were younger than 18 years (*n* = 8), yielding a study population over the 4-day period of 62 patients. Furthermore, to evaluate patients with thoracic aortic and mediastinal pathologies, a database search in the department’s radiology information system (RIS) was performed for patients with Stanford type A and/or B dissections, intramural hematomas, mediastinal lymphadenopathy, and pericardial effusions, or whose status was post thoracic endovascular aortic repair (TEVAR). For each of these pathologies or procedures, the corresponding last 10 patients (retrospectively from 14 February 2020) were retrospectively included in the study. Among the patients with clinically suspected dissection of the aorta, nine examinations were acquired with electrocardiogram (ECG) gating.

Out of the total 122 included patients, 93 underwent contrast-enhanced (arterial and/or venous phase) CT imaging, while 29 patients underwent non-contrast exams.

An additional sub-set consisting of 40 patients in total (20 male, 20 female), was formed to establish reference standards for the proximal arch, mid arch, and proximal descending aorta, respectively. A detailed description can be found in the Section 2.4.

### 2.2. Imaging Protocol

CT scans were acquired on a 3rd generation dual-energy CT system (2 × 192 slice SOMATOM Force, Siemens Healthcare, Forchheim, Germany) with the following parameters: automated tube voltage selection 90–150 kVp (CARE kV, Siemens, Erlangen, Germany) and scanogram-based automated tube current modulation (CARE Dose4D, quality reference mAs: 75–294); table speed 46–691.2 mm/s, table feed per rotation 23–172.8 mm, spiral pitch factor 0.6–3; dose length product (DLP) 12–1180.4 mGycm; collimation width 38.4–57.6 × 0.6 mm with z-flying focal spot. Dual-source mode was implemented on 26 patients. Images were reconstructed, with a slice thickness of 3.0 mm, increment of 3.0 mm, and various reconstruction kernels (Bf40, Bf44, Br40, Bv40, or Bv44). Iterative reconstruction was used with ADMIRE strength level 2. If required, a contrast agent was administered intravenously, adapted to the patient’s body weight (400 mg iodine per kg body weight; Imeron 400 [Iomeprol], Bracco Imaging, Konstanz, Germany) with an automated double syringe power injector and followed by a 30 mL saline flush; arterial and portal venous phase were acquired with a fixed delay of 40 and 80 s after contrast injection, respectively. The flow rate was 1.5–3.0 mL/s depending on the indication of the CT as well as the gauge of the peripheral line.

### 2.3. Algorithm

**Figure 1:**
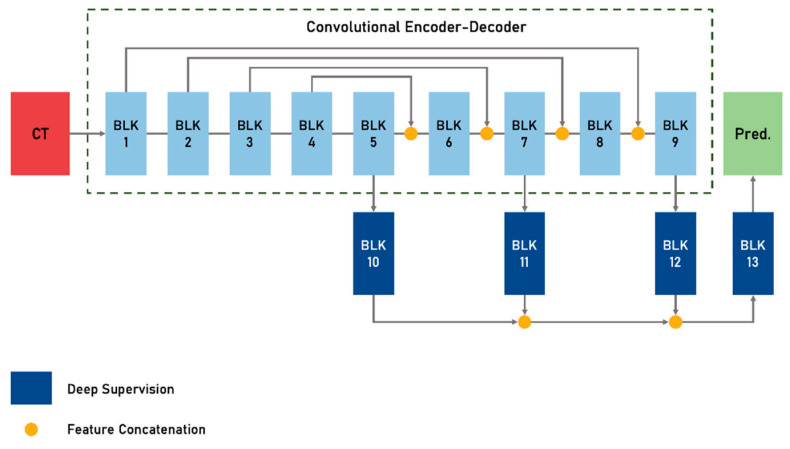
Deep image-to-image network (DI2IN). The front part is a convolutional encoder-decoder network with feature concatenation, and the backend is a deep supervision network through multi-level. Blocks inside DI2IN consist of convolutional and upscaling layers.

The thoracic aorta was segmented via a fully automated algorithm based on a deep adversarial image-to-image network, while identification of the measurement locations was conducted via deep reinforcement learning. This means in more detail: Aortic landmarks (Aortic Root/Aortic Arch Center/Brachiocephalic Artery Bifurcation/Left Common Carotid Artery/Left Subclavian Artery/Celiac Trunk) are detected automatically based on Deep Reinforcement Learning [4,11]. The aortic root is used to define an ROI for the segmentation algorithm. Within the ROI the segmentation is performed using an adversarial deep image-to-image network (DI2IN) in a symmetric convolutional encoder-decoder architecture (Figure 1) [12]. Given the aorta mask, a centerline model is used to generate the aortic centerline.

**Figure 2:**
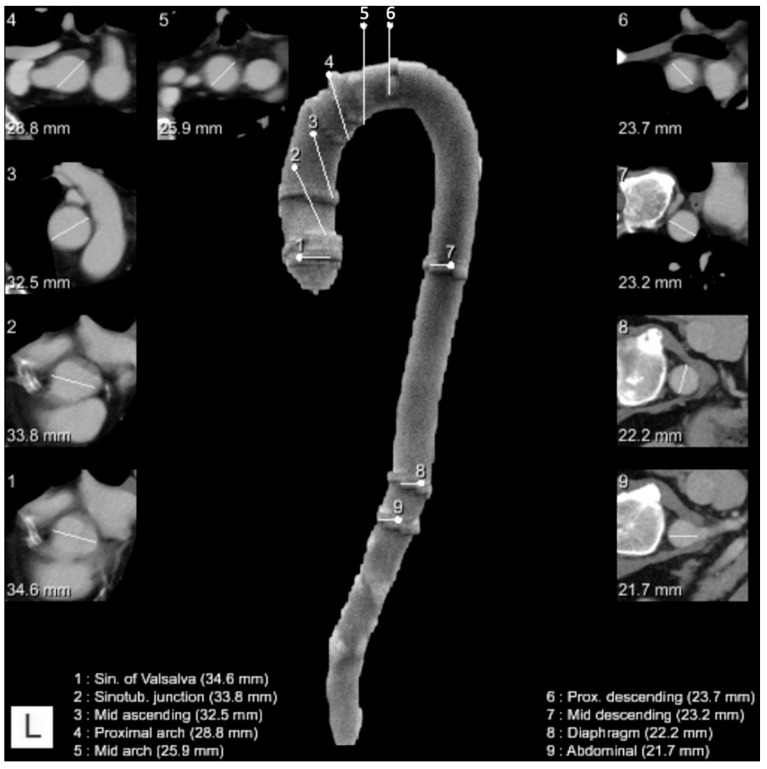
Example of a reconstruction of the thoracic aorta with location and diameter of the measurements at each dedicated location (legend on the bottom of the image describing the location and the measured diameter). White lines: measurements of the diameter at each dedicated location. In frame images (numbered 1–9 in the top left corner): depiction of the automated diameter measurements in the respective planes perpendicular to the center line.

**Figure 3:**
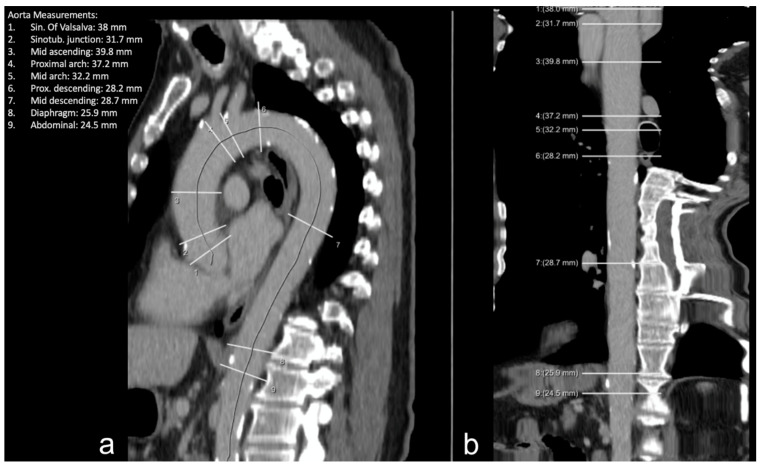
Example of the thoracic aorta imaged via CT in plane reformatted along the center line (**a**) and straightened reconstruction (**b**). White lines: measurement planes of the diameter at each dedicated location.

After the segmentation of the aorta, the algorithm computed the center line and detected nine anatomical locations defined according to the 2010 AHA guidelines: the sinus of Valsalva, sinotubular junction, mid-ascending aorta, proximal aortic arch, mid-aortic arch, proximal descending aorta, mid-descending aorta, aortic hiatus, and abdominal supraceliac aorta [13]. In each of the measurement planes, multiple diameters are computed by computing intersections of rays starting from the centerline with the aortic mask. Based on these diameters, the maximum in-plane diameter and the diameter along a direction orthogonal to the maximal diameter are finally provided by the algorithm. So, for each location, the largest aortic diameter in the respective plane perpendicular to the center line was measured automatically and reported (Figure 2 and Figure 3). This approach was applied to both contrast-enhanced and non-contrast enhanced CT scans without the need to use ECG gating.

This algorithm is embedded in the AI-Rad Companion Chest CT software (Siemens Healthineers, Erlangen, Germany) and was evaluated here as a prototype version. The software did not include measurements of the infraceliac abdominal aorta (Figure 2 and Figure 3).

### 2.4. Data Analysis

The department’s clinical post-processing three-dimensional viewer “*syngo*.via” (Version: VB30A_HF06; Siemens Healthcare, Erlangen, Germany) was used to remeasure all datasets at the abovementioned anatomical locations by two radiologists with 5 and 8 years of experience in thoracic imaging, respectively. Cases were evaluated in a blinded (to the patients’ medical history and clinical indication of the CT, the measurements of the algorithm, and the other reader) and randomized fashion. Using the measured values of both radiologists at each dedicated location, the mean diameter was determined, which was then compared to the measured diameter of the prototype.

The aorta was defined as being aneurysmatic if the diameter increased the average normal diameter by 150% (Table 1). Since there were no normal reference diameters for the proximal arch, mid arch, and proximal descending aorta in the literature, a sub-collective of 40 patients in total, consisting of 20 males (Range: 18–84 years) and 20 females (Range: 20–84 years), respectively, was established to determine reference diameters at these locations. CT scans for subjects in this sub-collective were conducted for non-cardiovascular reasons.

### 2.5. Statistical Analysis

The acquired data were analyzed using JMP 14.2.0 (SAS Institute). Continuous values are listed as mean ± standard deviation (SD). Relative frequencies are given as *n* (%). Equivalency was tested using the Wilcoxon paired test with Bonferroni correction accounting for multiple testing. Inter-reader agreement was assessed using intra-class correlation (ICC), which was defined as follows: poor (≤0.5), moderate (0.51–0.75), good (0.76–0.90). and excellent (>0.90) [14]. Furthermore, a Bland–Altman plot was generated for each dedicated anatomical location to visualize and compare measurements and errors between the algorithm and the readers. Statistical significance was set at a *p*-value of 0.05.

## 3. Results

In total, 122 patients (mean age: 60.4 ± 16.1 years; range: 18–94 years; 54.1% male) were included in the analysis. In 20.5% of the cases, the thoracic aorta had at least one aneurysmal segment. The area with the highest prevalence of aneurysmatic dilatations was the ascending part of the thoracic aorta, seen in 9.8% of subjects. Detailed demographic and subject data are shown in Table 2.

Among all measured parameters, 99.2% were successfully assessed by the prototypical segmentation tool. In nine patients, the segmentation tool failed to measure one diameter along the vessel (diameter of the mid-ascending aorta for eight patients [88.9%], diameter of the mid-descending aorta for one patient [11.1%]).

In patients with Stanford type A and/or B dissections, the segmentation tool measured the true and false lumen together, if both had blood flow. If the false lumen was thrombosed, only the true lumen was measured by the algorithm. The remaining pathologies or post-procedural states included in this study (post-TEVAR status, intramural hematoma, mediastinal lymphadenopathy, and pericardial effusion) did not lead to erroneous measurements of the prototypical segmentation tool.

**Figure 4:**
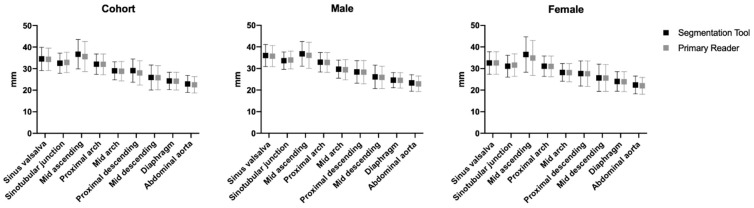
Mean measurements of the segmentation tool and the reader at the respective locations of the thoracic and abdominal supraceliac aorta. Data are presented in mm ± SD.

**Figure 5:**
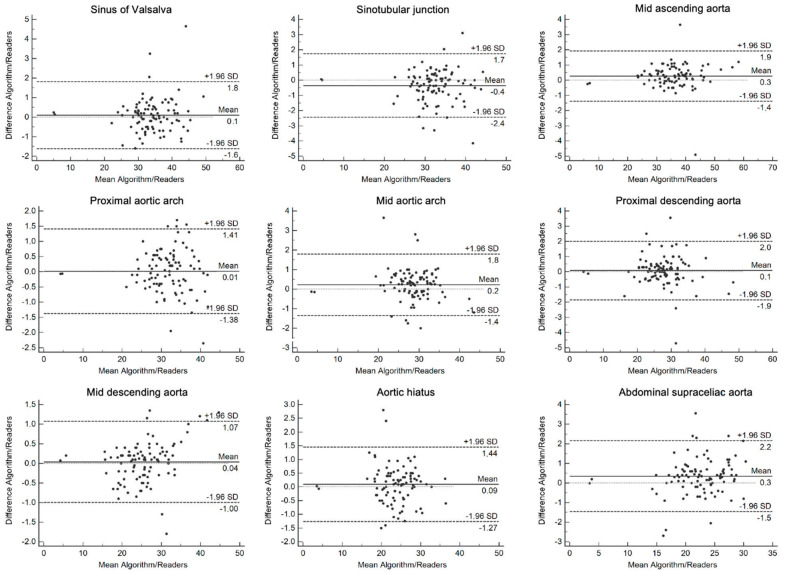
Bland–Altman plots demonstrating the mean bias (solid black line), and upper and lower limits of agreement (dashed black lines) for each measured location by the algorithm and readers along the thoracic aorta. The x- and y-axis are given in millimeters (mm).

The mean values of the measured diameters of each segment for the segmentation tool and the readers are shown in Figure 4 and Supplementary Table S1. Comparing the measurements for each segment along the thoracic aorta, there was no significant difference between the segmentation tool and the radiologist readers (*p* > 0.05), establishing equivalence (see also Figure 5). The largest difference in measurement of the diameter between the segmentation tool and the readers was seen in the sinus of Valsalva with a diameter measurement of 41.8 mm and proximal descending aorta with a diameter measurement of 33.7 mm (discrepancy: 4.7 mm, respectively), as well as mid-ascending aorta with a diameter measurement of 45.8 mm (discrepancy: 4.9 mm), both of which were measured in patients without any pathology (Figure 5). Among the patients with pathologies, the largest discrepancy between the algorithm and the readers was observed at the proximal descending aorta with 3.9 mm.

The measurements between the contrast-enhanced and unenhanced datasets did not significantly differ (*p* > 0.05).

Inter-reader agreement between the algorithm and the mean of the radiologists was excellent for each dedicated location with an ICC between 0.961 (95% CI: 0.940–0.974) in the mid-ascending aorta and 0.984 (95% CI: 0.977–0.990) in the mid-descending aorta.

Inter-reader agreement between the two radiologists was excellent for each measured location of the aorta with an ICC of ≥ 0.879 (95% CI: 0.818–0.920). The lowest level of agreement was observed in the region of the proximal arch and the highest level of agreement at the mid-ascending aorta (ICC = 0.958; 95% CI: 0.937–0.972).

## 4. Discussion

This study evaluated the accuracy of a prototypical AI-based automated segmentation and measurement algorithm designed to determine the diameter of the thoracic aorta via CT. The algorithm can be applied to any CT of the thorax since it requires neither contrast nor ECG gating. The measured diameters along the dedicated segments of the thoracic and supraceliac aorta as determined by the prototypical algorithm were equivalent to the measurements of two independent readers, including the presence of aortic and mediastinal pathologies. This was also recently reported in a study by Monti et al. using the same prototypical algorithm [15]. Furthermore, this study’s inter-reader agreement of the manually acquired measurements along the thoracic aorta was excellent, with the lowest agreement in the proximal arch. Although this was still classified as excellent agreement, this small discrepancy may be due to the vascular outlet of the right subclavian artery, which may be partially included in the measurement depending on the slice of acquisition. The results of this study are in line with previously reported inter-reader agreements of ICC > 0.82, including studies that segmented abdominal aortic diameters [1,16,17,18,19,20]. However, compared to these studies, there was no observer bias regarding average diameters observed for the segmentation tool or the readers [1,21]; this is in contrast to a study by Asch et al. in which ICC decreased to 0.73–0.85 among the thoracic segments of the aorta when measurements were compared between clinical centers and core laboratories with standardized measuring protocols [22,23].

The diameters along the thoracic aorta measured by the prototypical algorithm and the radiologists were comparable to previous studies, including differences observed between genders. For example, the most affected segment of the thoracic aorta, the ascending part, reported diameters in the literature were 36.7 to 37.1 ± 3.5 to 4.0 mm for male and 31.4 to 34.5 ± 3.3 to 4.0 mm for female patients, with the prototype measurements of this study being at the lower end for males (36.8 ± 5.7 mm) and upper end for females (36.5 ± 8.2 mm) of this spectrum [1,24,25]. The remaining segments of the thoracic aorta demonstrated similar results when comparing diameter measurements [26,27]. The slight differences in the measurements may be due to specific CT protocols, variation within the studied population, and/or the algorithm itself [1]. In addition, differences in the specific planes and slice thicknesses used to acquire the vessels’ diameter may introduce variance to the measurements between readers and studies [28].

In this study, the segmentation tool was used both with contrast-enhanced (arterial or portal venous) and unenhanced CT images of the thorax. The majority of studies so far have used contrast-enhanced CT angiography to assess the automated segmentation of the aorta; consequently, the lumen, and thus the border of the vessel, is much more distinctive as compared to unenhanced datasets [17,29,30,31,32]. Some studies have performed the segmentation with unenhanced images [33,34,35], such as Sedghi Gamechi et al. who evaluated the diameters of the thoracic aorta on a fully automated algorithm on 1334 unenhanced and non-ECG-gated CT scans. Due to the reliability of the algorithm, they concluded that such a tool may be promising for screening in clinical routine [1].

Due to the fully automated nature of the AI-based segmentation tool, it may facilitate more rapid reporting times by up to 63% and increase inter-reader agreement in the clinical setting, especially for care involving multiple clinical centers. In addition, since the diameter of the aorta increases with age, male sex, and body surface area [13,24,36,37], further development of the algorithm to include this pertinent patient information in the analysis of a potentially aneurysmatic diameter of the aorta could further enhance its clinical utility.

This study has several limitations. First, CT scans of the thorax were not ECG-gated, which is not necessary for determining aneurysms of the thoracic aorta or in non-vascular specific scans; however, it may result in erroneous measurements in the sinus of Valsalva and the sinotubular junction (aortic root). This may be a particular issue in patients with Marfan’s syndrome, who can rarely have an isolated aneurysm of the aortic root [1,38]. Although the ascending aorta may be impacted by motion artifacts, measured diameters may still be fairly accurate; this will be a topic of future study. Secondly, due to the small sample size, the validity for patients with TAA is limited. Although the few patients with TAA or above-described pathologies were segmented correctly by the tested segmentation tool, further investigation leveraging larger, disease-specific cohorts will be required. Thirdly, a randomly selected cohort that included unenhanced and enhanced datasets seems to have been correctly assessed by the segmentation tool; however, further studies in specific patient cohorts with standardized protocols are necessary. Fourth, there is growing evidence that the total length of the aorta has an impact on aortic pathologies, such as dissections [39]. Therefore, the addition of measurement of aortic length, as well as an independent measurement of the true and false lumen diameter, may further enhance the evaluation of aortic pathologies. Lastly, the observed inter-reader agreement of this study may be influenced by the single-center approach.

In conclusion, the evaluated prototypical AI-based algorithm accurately measured thoracic aortic diameters at each of the regions of interest independent of the use of either contrast utilization or pathology. These results indicate that the prototypical algorithm has substantial potential as a valuable tool in the rapid clinical evaluation of aortic pathology.

## Figures and Tables

**Table 1:** Non-pathological and aneurysmatic thoracic aortic diameters in adults (reported in millimeters), rounded to the nearest millimeter [11]. * Values of normal and aneurysmatic thoracic aortic diameters established using data from the sub-collective.

Thoracic Aorta	Male		Female	
	Normal	Aneurysm	Normal	Aneurysm
Aortic root (Sinus Valsalva and Sintotublar Junction)	39	59	37	56
Ascending aorta	29	44	29	44
Proximal arch	30 *	45 *	28 *	42 *
Mid arch	27 *	41 *	25 *	38 *
Proximal descending	26 *	39 *	24 *	36 *
Mid-descending	30	45	26	39
Diaphragmatic	27	41	25	38
Supracoeliac Abdominal	30	45	27	41

**Table 2:** Demographic and subject data. The remaining locations of the aorta are not mentioned due to no found aneurysms.

	Cohort	Male	Female
Age (years)	69.4 ± 16.1	58.7 ±16.8	62.4 ± 15.1
*n* (%)	122	66 (54.1%)	56 (45.9%)
Aneurysm *n* (%)	25 (20.5%)	11 (16.7%)	14 (25%)
Ascending aorta	12	7	5
Proximal arch	2	0	2
Mid arch	2	1	1
Proximal descending	6	3	3
Mid-descending	2	0	2
Diaphragm	1	0	1

## Data Availability

Not applicable.

## Supplementary Materials

The following supporting information can be downloaded at: https://www.mdpi.com/article/10.3390/diagnostics12081790/s1, Table S1: Mean measurements of the segmentation tool and the primary reader at the respected locations of the thoracic aorta. Data is given in mm ± SD.
