# Supplementary material for: Assessing the Accuracy of an Artificial Intelligence-Based Segmentation Algorithm for the Thoracic Aorta in Computed Tomography Applications
Source: Diagnostics (Basel). 2022 Jul 23;12(8):1790. doi: 10.3390/diagnostics12081790 (PMC9330011; doi:10.3390/diagnostics12081790)
Supplement: Supplementary file 1 [file diagnostics-12-01790-s001.zip › diagnostics-1718160-supplementary.pdf]

**Supplementary Materials:**

**Table S1.** Mean measurements of the segmentation tool and the primary reader at the respected locations of the thoracic aorta. Data is given in mm ± SD.

| Thoracic aorta | Segmentation Tool | | | Primary Reader | | |
|---|---|---|---|---|---|---|
| | Cohort | Male | Female | Cohort | Male | Female |
| Sinus valsalva | 34.5 ± 5.4 | 36.0 ± 5.1 | 32.6 ± 5.2 | 34.3 ± 5.2 | 35.7 ± 4.9 | 32.6 ± 5.2 |
| Sinotubular junction | 32.5 ± 4.7 | 33.6 ± 4.0 | 31.1 ± 5.1 | 32.9 ± 4.7 | 33.9 ± 4.1 | 31.6 ± 5.2 |
| Mid ascending | 36.7 ± 6.8 | 36.8 ± 5.7 | 36.5 ± 8.2 | 35.6 ± 7.0 | 36.1 ± 6.0 | 34.9 ± 8.1 |
| Proximal arch | 32.1 ± 4.7 | 32.9 ± 4.5 | 31.1 ± 4.7 | 32.0 ± 4.8 | 32.8 ± 4.6 | 31.0 ± 4.8 |
| Mid arch | 29.0 ± 4.2 | 29.7 ± 4.2 | 28.2 ± 4.1 | 28.8 ± 4.5 | 29.4 ± 4.7 | 28.1 ± 4.2 |
| Proximal descending | 29.1 ± 5.4 | 28.4 ± 5.2 | 27.7 ± 5.8 | 28.0 ± 5.6 | 28.3 ± 5.4 | 27.6 ± 5.9 |
| Mid descending | 25.9 ± 5.8 | 26.1 ± 5.2 | 25.7 ± 6.3 | 25.8 ± 5.6 | 25.9 ± 5.2 | 25.6 ± 6.2 |
| Diaphragm | 24.3 ± 4.0 | 24.6 ± 3.5 | 24.0 ± 4.5 | 24.2 ± 4.1 | 24.5 ± 3.6 | 23.9 ± 4.6 |
| Abdominal aorta | 22.9 ± 3.9 | 23.3 ± 3.8 | 22.4 ± 4.1 | 22.5 ± 3.8 | 22.9 ± 3.6 | 22.0 ± 3.9 |
